# The Impact of Dietary Unsaturated Fat or the Mediterranean Diet on Women Diagnosed With Breast Cancer: A Systematic Review

**DOI:** 10.7759/cureus.65362

**Published:** 2024-07-25

**Authors:** Shikha Virani, Sumayya Afreen, Arvin Perthiani, Elizabeth Sangster, Nidhi Lanka, Prakash Acharya, Ann K Yu

**Affiliations:** 1 Internal Medicine, California Institute of Behavioral Neurosciences & Psychology, Fairfield, USA; 2 Medicine, Deccan College of Medical Sciences, Hyderabad, IND; 3 Obstetrics and Gynecology, California Institute of Behavioral Neurosciences & Psychology, Fairfield, USA; 4 General Surgery, Our Lady of Lourdes Hospital, Louth, IRL; 5 Medicine, Saint George's University, St. George, GRD; 6 Psychiatry and Behavioral Sciences, California Institute of Behavioral Neurosciences & Psychology, Fairfield, USA; 7 General Medicine, California Institute of Behavioral Neurosciences & Psychology, Fairfield, USA

**Keywords:** plant-based diet, anti-inflammatory diet, antioxidants, polyunsaturated fatty acids, omega-3 fatty acids, quality of life, cancer survival, unsaturated fats, mediterranean diet, breast cancer

## Abstract

This review explores the multifaceted relationship between dietary factors and breast cancer outcomes, focusing on unsaturated fats, the Mediterranean diet (MD), and other nutritional components. Breast cancer remains a significant global health concern, with lifestyle factors like diet playing a pivotal role in prevention and management. The review adhered to the Preferred Reporting Items for Systematic Reviews and Meta-Analyses (PRISMA) 2020 guidelines. Articles written in English and released between 2019 and 2024 were acceptable. We used pertinent search terms such as "unsaturated fats", "Mediterranean diet", "breast cancer", and "nutrition" to perform searches in PubMed, PubMed Central (PMC), EBSCOhost, and grey literature such as Google Scholar. After screening, 11 of the 479 original papers were chosen and included in the final review. These include cross-sectional analysis and systematic review, cohort study, narrative review, systematic review and meta-analysis, case-control study, randomized controlled trials (RCTs), and cross-sectional study. Key findings suggest that adherence to the MD correlates with improved quality of life measures and reduced mortality rates among women with breast cancer, particularly in older age groups. The diet's emphasis on antioxidant-rich foods, anti-inflammatory compounds, and healthy fats contributes to these observed benefits. Specific unsaturated fats, notably omega-3 polyunsaturated fatty acids (PUFAs) like docosahexaenoic acid (DHA) and eicosapentaenoic acid (EPA), demonstrate anti-cancer properties by modulating cancer cell behavior and enhancing treatment responses. Biomarkers associated with the MD, such as β-carotene and lycopene, serve as indicators of dietary compliance and potential risk reduction. Furthermore, components found in olive oil, including polyphenols and monounsaturated fatty acids, exhibit promising effects in preventing breast cancer by exerting antioxidant and anti-proliferative actions. Other dietary factors like calcium, legumes, fruits, and vegetables also play a role in reducing breast cancer risk and improving survival rates. This review underscores the importance of dietary interventions in optimizing outcomes for breast cancer patients and highlights the need for further research to elucidate underlying mechanisms and refine dietary recommendations.

## Introduction and background

Breast cancer is a condition in which malignant breast cells proliferate uncontrollably and develop into tumors. The risk is highest in females and affects women at any age after adolescence; however, its prevalence rises with age. It is estimated that women account for 99% of breast cancer cases, whereas men account for 0.5-1% of cases. In the year 2022, 2.3 million women worldwide were diagnosed with breast cancer, and 670,000 died from the disease [1]. Ninety-five percent of breast cancer cases are sporadic and not inherited [2]. It has a 99% five-year relative survival rate when detected in its early and localized stages. In the United States (US), there are currently more than four million breast cancer survivors because of significant advancements in early diagnosis and treatment methods in recent years [3].

In the US, breast cancer is the second most prevalent cancer among women (certain types of skin cancer are the most frequent). Compared to white women, black women experience a greater death rate from breast cancer [4]. More than 151,000 breast cancer patients were included in the 108 international trials that the researchers examined, and review findings suggest that increasing intake of dietary fiber from fruits, vegetables, pulses, and whole grains may increase survival. Additionally, there was some evidence to support the idea that consuming soy might lower the chance of dying or developing breast cancer again. Soy foods include tofu, tempeh, edamame, and soy drinks [5]. Furthermore, several leafy green veggies that may possess anticancer qualities include kale, mustard greens, chard, arugula, and spinach. Leafy green foods have carotenoid antioxidants, such as beta-carotene, lutein, and zeaxanthin. Elevated blood concentrations of these antioxidants are linked to a decreased risk of breast cancer [6]. 

The Mediterranean diet (MD) is a plant-based dietary pattern characterized by a low to moderate intake of dairy products, fish, and poultry, a moderate intake of alcohol, and a low intake of sweets and red meat. Plant foods include fruits, vegetables, legumes, nuts, and unrefined cereals [7]. Unsaturated fats come in two varieties: monounsaturated and polyunsaturated. Avocados, nuts, seeds, olives, and olive oils are good sources of monounsaturated fats. Polyunsaturated fats (PUFAs) are present in walnuts, flaxseeds, soybean, corn, sunflower, and soybean oils. Two important fats, omega-3 and omega-6 fatty acids, are also found in PUFAs [8]. Fruits, nuts, olive oil, and plant-based meals release antioxidants, polyphenols, and monounsaturated fatty acids that may have a beneficial effect on pain, inflammation, and endothelial function [9]. Many mechanisms, including weight management, antioxidant capacity, improved glycemic profile, and anti-inflammatory activities, are likely to be responsible for the beneficial health effects attributed to the MD in patients diagnosed with breast cancer. The MD has been associated with a lower incidence of breast cancer, but definitive data from prospective trials regarding the diet's effect on breast cancer survival is still lacking [10]. Therefore, this systematic review aims to identify and summarize the impact of dietary unsaturated fats or the Mediterranean diet on women diagnosed with breast cancer. 

## Review

Methods

Guidelines

The Preferred Reporting Items for Systematic Reviews and Meta-Analyses (PRISMA) 2020 standards were followed for the reporting [7]. The PICO strategy and review question focused on the population (P) of women diagnosed with breast cancer. The intervention (I) examined the benefits of an unsaturated diet or the Mediterranean diet, and the outcomes (O) considered were recurrence rates, survival rates, and quality of life assessments. The central review question is "What potential health benefits may an unsaturated or Mediterranean diet provide for breast cancer patients?". 

Eligibility Criteria

We thoroughly searched databases (PubMed, PubMed Central (PMC), EBSCOhost) and grey literature (Google Scholar). Key terms for the search were "unsaturated dietary fat", "Mediterranean diet", "plant-based diet", "healthy fat diet", "low-fat diet", "olive oil-rich diet", "DASH diet", "breast cancer", "mammary carcinoma", "breast carcinoma", "breast tumor", "breast neoplasm", "mammary neoplasm", "malignant breast disease", "breast malignancy", "mammary cancer", "carcinoma of the breast", "women", and "breast adenocarcinoma". Medical Subject Heading (MeSH) keywords were located for use with Boolean operators such as "OR" and "AND" in PubMed. A total of 479 papers were discovered by applying filters such as publications published between 2019-2024, written in English, and free-full text (Table 1). Following the transfer of all references to Excel (Microsoft, Redmond, Washington), any duplicate entries were subsequently eliminated. Eleven publications were included after each was reviewed separately. The studies were included following the evaluation of the quality assessment: one cross-sectional study and systematic review, one cohort study, one narrative review, two systematic reviews and meta-analyses, one case-control, four randomized controlled trials, and one cross-sectional study.

**Table 1:** Search strategy and results for dietary fats and breast cancer studies MeSH - Medical Subject Headings; DASH - Dietary Approaches to Stop Hypertension; PMC - PubMed Central; EBSCO - Elton B. Stephens Company

Database/registers	Search strategy	Filter applied	Results
PubMed	The MeSH search strategy "Unsaturated dietary fat" OR "Mediterranean diet" OR "Plant-based diet" OR "Healthy fat diet" OR "Low-fat diet" OR "Olive oil-rich diet" OR "DASH diet" OR "Dietary Fats, Unsaturated/administration and dosage"[Majr] OR "Dietary Fats, Unsaturated/blood"[Majr] OR "Dietary Fats, Unsaturated/classification"[Majr] OR "Dietary Fats, Unsaturated/history"[Majr] OR "Dietary Fats, Unsaturated/immunology"[Majr] OR "Dietary Fats, Unsaturated/metabolism"[Majr] OR "Dietary Fats, Unsaturated/pharmacokinetics"[Majr] OR "Dietary Fats, Unsaturated/pharmacology"[Majr] OR "Dietary Fats, Unsaturated/supply and distribution"[Majr] OR "Dietary Fats, Unsaturated/therapeutic use"[Majr] AND "Breast cancer” OR Mammary carcinoma OR Breast carcinoma OR Breast tumor OR Breast neoplasm OR Mammary neoplasm OR Malignant breast disease OR Breast malignancy OR Mammary cancer OR Carcinoma of the breast OR Breast adenocarcinoma OR "Breast Neoplasms/diet therapy"[Majr] OR "Breast Neoplasms/etiology"[Majr] OR "Breast Neoplasms/physiopathology"[Majr] OR "Breast Neoplasms/prevention and control"[Majr] OR "Breast Neoplasms/rehabilitation"[Majr].	Related to topic, 2019-2024 data, Free full text, English language.	173
Google Scholar	"Unsaturated dietary fat" OR "Mediterranean diet" AND "Breast adenocarcinoma" AND "Women"	2019-2024 data, Free full text, English language.	118
PMC	"Unsaturated dietary fat" OR "Plant-based diet" OR "Mediterranean diet" AND "Carcinoma of the breast"	Open access articles, English language, 2019-2024 data	110
EBSCOhost	"Plant-based diet" AND "Breast cancer" AND "Women"	EBSCO Open Research with Full Text, English language, Peer Reviewed, Full Text articles, Past 12 months data.	78

Data Analysis and Synthesis

Quality assessment: The quality of the included studies was rigorously assessed using established tools to ensure the reliability and validity of the findings. The Cochrane collaboration's risk of bias assessment technique for randomized controlled trials (RCTs) was employed, and five domains were examined to search for biases in each of the four studies. Each domain was assessed for risk as high, low, and unclear. To ensure rigorousness, only studies with a low risk of bias in at least two out of five domains were included in the systematic review. A detailed overview of research study characteristics, including the publication year, study design, quality appraisal tool used, funding, and number of references, are demonstrated in Table 2.

**Table 2:** Detailed overview of research study characteristics SANRA - Scale for the assessment of non-systematic review articles; PRISMA - Preferred Reporting Items for Systematic Reviews and Meta-Analyses; RCT - randomized controlled trials

Study citation	Year of publication	Study design	Quality appraisal tool used	Funding	References
Barchitta et al., 2020 [11]	2020	Cross-sectional analysis and systematic review	Newcastle-Ottawa Quality Assessment Scale	No	58
Di Maso et al., 2020 [12]	2020	Cohort study	Newcastle-Ottawa Quality Assessment Scale	Yes	32
Flore et al., 2023 [13]	2023	Narrative review	SANRA	No	304
Giordano et al., 2020 [14]	2020	Systematic review and meta-analysis	PRISMA	Yes	100
Heidari et al., 2020 [15]	2020	Case-control study	Newcastle-Ottawa Quality Assessment Scale	Yes	32
Long Parma et al., 2022 [16]	2022	RCTs	Cochrane Collaboration's Risk of Bias Tool	Yes	48
Montagnese et al., 2020 [17]	2021	RCTs	Cochrane Collaboration's Risk of Bias Tool	Yes	51
Negrati et al., 2021 [18]	2021	Cross-sectional study	Newcastle-Ottawa Quality Assessment Scale	No	53
Theinel et al., 2023 [19]	2023	Systematic review and meta-analysis	PRISMA	Yes	78
Rein et al., 2022 [20]	2022	RCTs	Cochrane Collaboration's Risk of Bias Tool	Yes	35
Tawfik et al., 2023 [21]	2023	RCTs	Cochrane Collaboration's Risk of Bias Tool	Yes	38

For cross-sectional analysis and systematic review, cohort, and case-control studies, the Newcastle-Ottawa Scale (NOS) was employed. These assessments ensured a rigorous evaluation of study quality. Each study was rated on a scale from zero to seven points. Studies achieving a score of at least five out of seven (>= 70%) were deemed acceptable for inclusion in the review. The NOS criteria for cross-sectional analysis, cohort study, and case-control study were demonstrated in Tables 3, 4, 5, respectively.

**Table 3:** Newcastle-Ottawa Quality Assessment Scale criteria for cross-sectional analysis

Newcastle-Ottawa Quality assessment scale criteria for cross-sectional analysis	Representativeness of the sample	Sample size	Non-respondents	Risk factor	Comparability	Assessment of the outcome	Statistical test	Total
Barchitta et al., 2020 [11]	*	*	-	**	*	**	*	6/7
Negrati et al., 2021 [18]	*	-	-	*	*	*	*	5/7

**Table 4:** Newcastle-Ottawa Scale criteria for cohort study

Newcastle-Ottawa scale criteria for cohort study	Representativeness of the exposed Cohort	Selection of the non-exposed cohort	Ascertainment of exposure	Demonstration that outcome of interest was not present at start of study	Comparability	Assessment of outcome	Was follow-up long enough for outcomes to occur	Adequacy of follow-up of cohorts	Total
Di Maso et al., 2020 [12]	*	*	*	*	**	*	*	*	8/8

**Table 5:** Newcastle-Ottawa Scale criteria for case-control study

Newcastle-Ottawa scale criteria for case-control study	Is the case definition adequate?	Representativeness of the cases	Selection of controls	Definition of controls	Comparability	Ascertainment of exposure	Non-response rate	Total
Heidari et al., 2020 [15]	*	*	-	*	**	*	-	5/7

One included narrative review underwent thorough quality assessment using the Scale for the Assessment of Non-systematic Review Articles (SANRA), which evaluates six items: justification of the article's importance, formulation of the question, description of literature search, referencing, scientific reasoning, and appropriate presentation of data. Each item was assessed for quality by scoring (0=low quality; 1=medium quality; 2=high quality). Study achieving a score of at least 9/12 (equivalent to 75%) was acceptable. The summary of the quality assessment using the SANRA checklist for narrative reviews can be seen in Table 6.

**Table 6:** SANRA checklist for narrative review SANRA - scale for the assessment of non-systematic review articles 0=low quality; 1=medium quality; 2=high quality

SANRA checklist	Justification of the article's importance	Formulation of question	Description of literature search	Referencing	Scientific reasoning	Appropriate presentation of data	Total
Flore et al., 2023 [13]	2	2	2	2	2	1	11/12

*Data Extraction* 

After removing duplicate studies, the first author narrowed down the research by using inclusion and exclusion criteria, reviewing abstract titles, and going over each report and quality appraisal application. March 2024, when PubMed and PubMed Central databases/registers were last used, while Google Scholar and EBSCOhost were last used in July 2024. Verification of abstract titles and use of quality rating methods were done independently by co-authors. By reaching a consensus and re-examining the relevant research, any conflicts were settled. A summary of various studies related to dietary interventions and outcomes measured in breast cancer patients, along with their respective limitations, are mentioned in Table 7.

**Table 7:** Summary of studies on dietary interventions and outcomes measured in breast cancer patients MD - Mediterranean diet; QoL - quality of life; BMI - body mass index; EORTC QLQ-C30 and QLQ-BR23 - European Organization for the Research and Treatment of Cancer Quality-of-Life Questionnaire and its Breast Cancer Module; MDS - Mediterranean diet score; DASH - Dietary Approaches to Stop Hypertension; RCTs - randomized controlled trials; PUFAs - polyunsaturated fatty acids

Study reference	Study design	Population	Interventions	Outcomes measured	Limitations
Barchitta et al., 2020 [11]	Cross-sectional study and systematic review	68 Italian stage I-III breast cancer survivors	Assessment of adherence to the MD, physical activity level, and QoL	Behavioral and dietary data; Anthropometric measures (e.g., BMI, QoL) as assessed by the EORTC QLQ-C30 and QLQ-BR23 questionnaires; Scores for various QoL domains, including emotional and cognitive functioning, insomnia, financial impact, and loss of appetite	Heterogeneity in study design, interventions, and tools for QoL assessment; lack of overall estimate from systematic review due to study differences
Di Maso et al., 2020 [12]	Cohort study	1453 women with breast cancer diagnosed between 1991-1994	Adherence to MD	Overall survival probabilities according to MDS; Cause-specific mortality; Hazard ratios of death for all causes; Differences in food groups or nutrient intake according to levels of adherence to the MD	Retrospective design; Potential dietary changes post-diagnosis; Lack of treatment data; Possible selection bias
Flore et al., 2023 [13]	Narrative review	Breast cancer survivors	Dietary interventions specifically the MD; Examination of nutraceuticals and functional foods particularly those rich in antioxidants, phenols, and other micronutrients	Potential effects on cancer recurrence, mortality, weight management;	Lack of data on dietary changes post-diagnosis; Absence of awareness and guidelines on diet-cancer links during the study period; Potential selection bias despite efforts to recruit representative breast cancer patients
Giordano et al., 2020 [14]	Systematic review and meta-analysis	Individuals with breast cancer	Anti-neoplastic effects of n−3 polyunsaturated fatty acid derivatives, specifically eicosapentaenoic acid (EPA) and docosahexaenoic acid (DHA) conjugates and amides	Detailed mechanisms of action including cell cycle arrest, apoptosis, anti-inflammatory effects of these derivatives on breast cancer cells	Limited to data from preclinical studies; Lacks clinical trial data; Potential bias in reviewed studies; Generalizability to clinical settings not fully established
Heidari et al., 2020 [15]	Hospital-based Case-control study	Women aged ≥30 years, breast cancer patients (cases) and women with non-neoplastic diseases (controls)	Assessment of dietary intake using a validated 168-item semi-quantitative food frequency questionnaire; Calculation of DASH index scores based on four established indexes (Dixon's, Mellen's, Fung's, and Günther's DASH diet indexes)	Evaluated odds ratios (OR) and 95% confidence intervals (CI) for breast cancer risk associated with different quintiles of DASH diet index scores	Recall bias due to the retrospective study design; Potential selection bias; Possibility of measurement errors in dietary assessments; Small sample size limited precision of results; Lack of information on breast cancer subtypes and hormone receptor status among cases
Long Parma et al., 2022 [16]	RCTs	153 overweight and obese (BMI ≥ 25 kg/m2), early-stage (0-III), English-speaking breast cancer survivors	Intervention group received monthly food-preparation workshops, motivational interviewing telephone calls, and tailored newsletters; Control group received informational brochures and non-motivational interviewing telephone calls	Mainly focused on QoL measures	Attrition of participants; Potential lack of sensitivity of QoL measures to behavioral interventions like dietary changes; Transient effect observed on perceived stress compared to other QoL indicators
Montagnese et al., 2021 [17]	RCTs	227 breast cancer survivors	Group 1: Low glycemic index MD + daily brisk walking + vitamin D supplementation. Group 2: Traditional MD + avoidance of physical inactivity + Vitamin D supplementation.	Health-related quality of life assessed	Potential biases associated with self-reported dietary and physical activity data; Generalizability limited to similar demographic and health profiles
Negrati et al., 2021 [18]	Cross-sectional study	80 women with a mean age of 54.9 ± 10.6 years (Histologically confirmed diagnosis of breast cancer and had not received pharmacological or radiotherapy treatment for at least two months)	Assessed for MDS, anthropometric parameters, and biomarker analysis including various blood parameters and micronutrient levels	Correlations between MDS and biomarkers	Limits causal inference and longitudinal assessment; Relatively small sample size; Relies on self-reported dietary habits through MDS questionnaire; Selection bias due to exclusion criteria and recruitment methods
Theinel et al., 2023 [19]	Systematic review	Animal models of breast cancer	Dietary enrichment with ω-3 PUFAs; Use of ω-3 PUFAs in the treatment or prevention of breast cancer	Effects of ω-3 PUFAs on breast cancer outcomes in animal models; Efficacy of ω-3 PUFAs supplementation in modifying tumor growth, metastasis, or response to other treatments in the context of breast cancer	Heterogeneity in experimental design, including variations in types of omega-3 PUFA used, doses, timing of supplementation relative to tumor induction; Inconsistent reporting of outcomes such as metastasis and survival
Rein et al., 2022 [20]	RCT	Hormone receptor-positive (HR+) patients with breast cancer (treated with adjuvant endocrine therapy)	Impact of an algorithm-based personalized diet versus a MD on weight and glycemic control	Impact of the dietary intervention on postprandial glycaemic response to meals, with a focus on weight and glycemic control	Single-blinded study design; Relies on daily use of smartphone application for dietary and lifestyle logging; Potential exclusion of patients without daily smartphone access
Tawfik et al., 2023 [21]	RCT	Adults (≥18 years), diagnosed with stage I-IV breast cancer	Treatment Group: Received 4 g/day of omega-3 acid ethyl esters (Lovaza) Control Group: Received placebo Timing: Started 1 week prior to paclitaxel treatment and continued until completion of paclitaxel	Primary Outcome: Incidence of paclitaxel-associated acute pain syndrome (P-APS); Secondary Outcomes: Pain severity scores, use of pain medications, incidence and severity of chemotherapy-induced peripheral neuropathy (CIPN)	Small sample size; High attrition rate (18%), Potential for type II error due to low statistical power; Compliance with supplementation regimen not fully assessed

Results

Search Outcome

**Figure 1:**
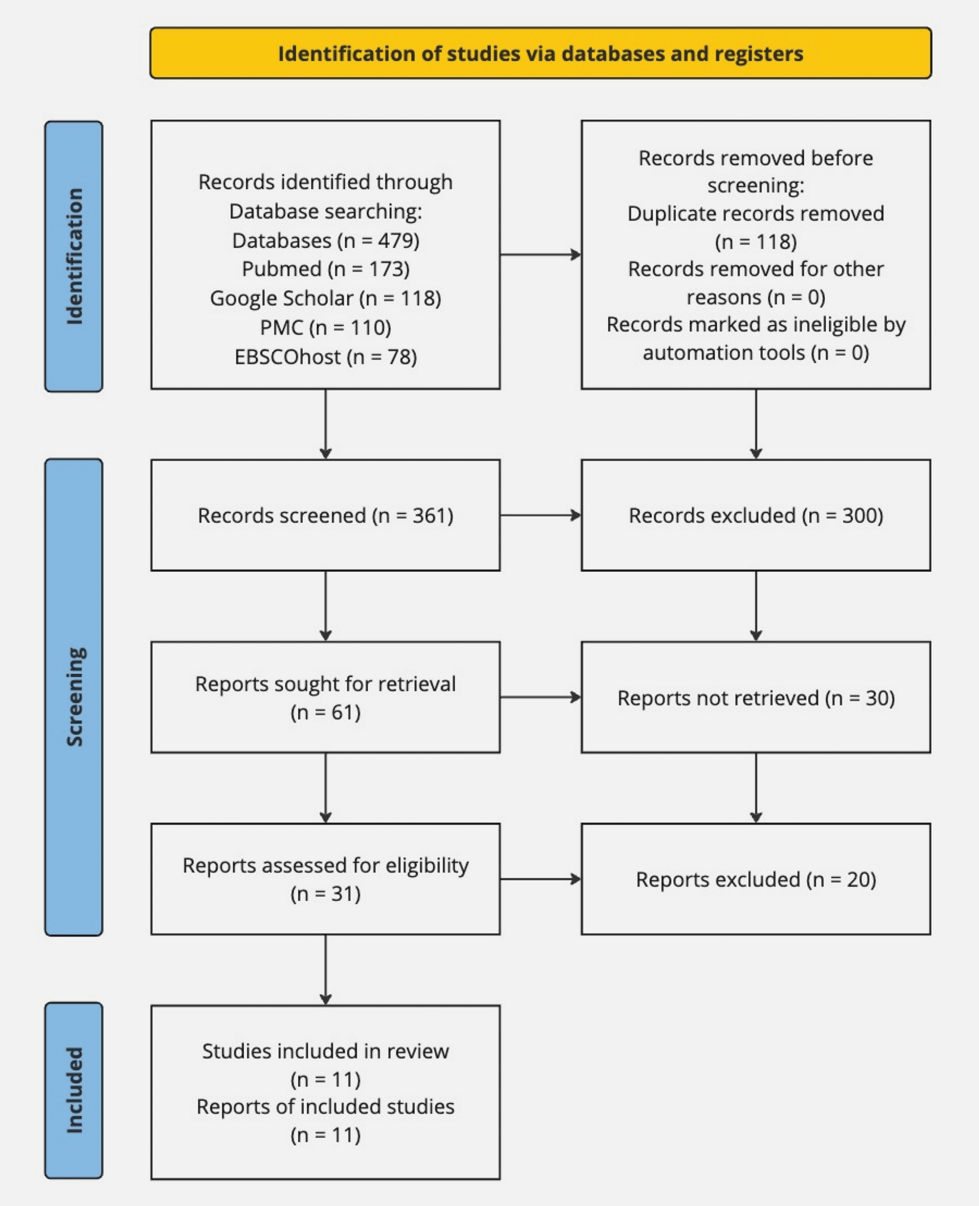
PRISMA flowchart of the study selection PRISMA - Preferred Reporting Items for Systematic Reviews and Meta-Analyses; PMC - PubMed Central

The initial literature search identified 479 articles from databases, including PubMed, PubMed Central, EBSCOhost, and grey literature (Google Scholar). After removing 118 duplicates, 361 articles were screened by the first co-author. From these, 300 were excluded due to irrelevance or inappropriate study design. Then, after the titles and abstracts of the records were used to evaluate them, a further 30 articles were excluded. Following the quality evaluation, 11 pertinent studies were chosen for the review, as shown in Figure 1.

Discussion 

Research on the effects of dietary choices, particularly those related to the consumption of unsaturated fats or the adoption of a Mediterranean diet (MD), on women who are diagnosed with breast cancer is a topic of great interest. The anti-inflammatory qualities of unsaturated fats, especially omega-3 polyunsaturated fatty acids (PUFAs), have been linked to possible advantages in the treatment and prevention of cancer. 

According to Barchitta et al., patients with breast cancer may benefit from following the MD, especially in terms of improved quality of life (QoL) results. Certain sub-scales of the MD, including minimal consumption of red meat, daily wine consumption, and high sofrito seasoning, were linked to improved quality of life ratings. Although further research is needed to determine the precise processes underlying these advantages, the MD's emphasis on eating a diet high in antioxidants, anti-inflammatory substances, and healthy fats may improve general health and even have an impact on cancer-related outcomes [11]. 

Di Maso et al. observed women with breast cancer may benefit from following a pre-diagnostic MD, especially in terms of survival rates. Strong MD adherence was linked to a lower incidence of deaths from all causes and causes other than breast cancer, with this association being particularly strong in women 55 years of age and older. The advantages of this diet are probably due to its constituents, which include high fiber, β-carotene from fruits and vegetables, and less animal fat. Still, the study found that among women under 55, the effect of the MD on survival was not statistically significant, possibly because these women typically have aggressive Breast cancer subtypes. Predictably, the MD's positive effect on breast cancer prognosis was stronger in patients who were overweight or obese, presumably because of its effect on comorbidities like metabolic syndrome. Despite significant limitations, such as the absence of post-diagnosis dietary data, the results indicate that the MD may be crucial in improving breast cancer patients' outcomes, particularly in specific demographic and health circumstances [12]. 

Based on Flore et al., these diets can lower the risk of death and recurrence in breast cancer patients when paired with consistent physical exercise. In particular, patients with breast cancer may have higher survival rates and better quality of life if they follow a diet low in meat and saturated fats and high in whole grains, fruits, vegetables, and healthy fats like those in olive oil. These eating habits have also been linked to decreased inflammatory levels, which is important for slowing the advancement of breast cancer. Furthermore, studies on breast cancer patients have demonstrated a substantial reduction in comorbidity, recurrence rates, and mortality while following the MD, which is well-known for its anti-inflammatory and antioxidant qualities. Overall, these diets have a good influence on patients' well-being and prognosis for breast cancer because of the synergistic effects of antioxidants, bioactive substances, and micronutrients [13]. 

Omega-3 Fatty Acids

Docosahexaenoic acid (DHA) and eicosapentaenoic acid (EPA) are two omega-3 polyunsaturated fatty acids (n−3 PUFAs) that have different mechanisms of action against breast cancer. They influence important signaling pathways by changing the composition of cell membranes and upsetting lipid rafts. They also cause apoptosis in cancer cells via decreasing inflammation, increasing oxidative stress, and modulating the activity of cyclooxygenase (COX). Furthermore, n−3 PUFAs influence the behavior of cancer cells by binding to receptors such as peroxisome proliferator-activated receptors (PPARs), transient receptor potential vanilloid 1 (TRVP1), G protein-coupled receptors (GPRs), and cannabinoid receptors (CBs). Their anti-cancer qualities are further enhanced upon conversion to bioactive derivatives, which makes them prospective medicines for the prevention and treatment of breast cancer. One important way that EPA and DHA prevent cancer is via binding to PPARγ, which is a peroxisome proliferator-activated receptor. The stimulation of apoptotic pathways in breast cancer cells has been connected to the activation of PPARγ, which helps to regulate the development of tumors. Further evidence for n-3 PUFAs' potential as therapeutic agents comes from studies that demonstrate how they function as direct agonists for PPARγ in breast cancer cells [14]. 

Legume

A weekly intake of one to two servings of legumes has been associated with a decreased risk of breast cancer; this association can be explained by the phytochemicals in legumes that impede cell growth and the fiber content that prevents estrogen from being circulated [22]. In contrast to women who consume beans or lentils less frequently than once a month, those who consume them twice a week or more have a significant 24% lower risk of developing breast cancer. Clearly, beans are an essential part of any diet designed to fight cancer [23]. 

Vegetables and Fruits

The antioxidant properties, fiber content, and vitamins C and E of fruits and vegetables reduce oxidative stress and shield DNA from damage, making them highly associated with a significantly lower risk of breast cancer when consumed in three to five servings per day [22]. Because fruits and vegetables differ significantly in composition, it is not expected that they will have comparable correlations with breast cancer survival. Specifically, berry consumption following diagnosis may contribute to increased survival. It has been demonstrated that berries and their bioactive components, such as their phenolic and anthocyanin contents, have anti-proliferative, anti-inflammatory, and anti-angiogenic qualities [24]. They may also slow the growth and metastasis of tumors, cause apoptosis in breast cancer cells, and prevent the progression of cancer. These results add to the growing corpus of in vitro and in vivo berry research [24,25]. 

Calcium

Dairy products with calcium have also been associated with a decreased risk of breast cancer, though the exact mechanism is still unknown. These findings were partially supported by a recent meta-analysis, which may have been influenced by the type and amount of fat in dairy products, growth hormones in milk, and environmental contaminants. Conjugated linoleic acid (CLA)-containing dairy products were believed to be chemo-protective agents, but there was considerable disagreement in the literature regarding the substance's capacity to prevent breast cancer [21,26]. 

Biomarkers and MD

There are important insights and useful advantages to the favorable link shown between β-carotene, lycopene, and following the Mediterranean diet. First, as greater levels of these biomarkers are linked to a lower risk of cancer, it suggests a possible reduction in cancer risk, particularly for breast cancer survivors. Furthermore, these biomarkers function as verifiers of dietary compliance, offering a concrete means of verifying if people are faithfully adhering to the Mediterranean diet. Additionally, tracking levels of lycopene and β-carotene can serve as a predictor of general health status and provide insight into future health outcomes for individuals, especially those who have survived cancer [18,27]. Furthermore, because oxidative stress and inflammation are associated with the onset and spread of cancer, the antioxidant and anti-inflammatory qualities of lycopene and β-carotene play a critical role in preventing them. This association provides access to customized nutrition, enabling people to modify their diets to incorporate more foods high in lycopene and β-carotene, thereby potentially improving their health outcomes, particularly in managing and preventing cancer [18]. 

PUFA in Addition to Other Anti-Tumor Therapies

The effects of ω-3 PUFA supplementation in combination with conventional anti-tumor treatments were the subject of a systematic review that revealed a range of results regarding mitosis inhibition, reduction of inflammation, pain relief, weight control, support for chemotherapy, and improvement of overall survival in patients with breast cancer. There are, however, contradictory findings about the direct influence of ω-3 PUFA on tumor therapy. According to certain studies, ω-3 PUFA can prevent angiogenesis, reduce inflammation, and inhibit growth in order to have anti-cancer effects. Standard recommendations exist regarding the consumption of ω-3 PUFA in various health contexts, with an emphasis on its potential benefits for improving cardiovascular health, pregnancy outcomes, and general well-being [19,28]. 

Olive Oil

Olive oil has anti-cancer properties due to the presence of squalene and monounsaturated fatty acids, such as oleic acid. Due to the presence of additional bioactive compounds like lignans, oleocanthal, oleuropein, and hydroxytyrosol, olive oil may help prevent breast cancer. Oleic acid and squalene have anti-proliferative and antioxidant qualities, respectively. In breast cancer cells, it has been demonstrated that polyphenols like hydroxytyrosol, oleocanthal, and oleuropein lessen oxidative stress, DNA damage, and tumor development. Lignans, being phytoestrogens, have been associated with a decreased risk of breast cancer in women who have undergone menopause. These findings suggest that the components of olive oil may have a protective effect against breast cancer [29]. 

One of the systematic review's strengths is the extensive search strategy it employed across several databases, covering a range of study designs to present an integrated picture. The inclusion of trustworthy studies with sound methodology is ensured by the use of high-quality appraisal tools. However, limitations such as potential publication bias, limited generalizability due to inclusion criteria, and incomplete data in some studies highlight areas for improvement. Addressing these limitations could enhance the overall reliability and applicability of the review's conclusions. Summary of studies on the impact of dietary patterns on breast cancer outcomes is mentioned in Table 4.

**Table 8:** Summary of studies on the impact of dietary patterns on breast cancer outcomes MD - Mediterranean diet; PUFA - polyunsaturated fatty acid; DHA - docosahexaenoic acid; EPA - eicosapentaenoic acid; RCT - randomized controlled trial

Study reference	Key findings
Barchitta et al., 2020 [11]	Adherence to the MD is correlated with improved quality of life measures among breast cancer patients. Specific MD components, like minimal red meat consumption and high sofrito seasoning, were associated with better quality of life ratings.
Di Maso et al., 2020 [12]	Strong adherence to MD was linked to lower all-cause mortality rates among breast cancer patients, especially in women aged 55 and older. MD's benefits were attributed to high fiber, β-carotene intake, and lower animal fat consumption.
Flore et al., 2023 [13]	Diets low in meat and saturated fats, high in whole grains, fruits, vegetables, and healthy fats (like olive oil) were associated with higher survival rates and a better quality of life among breast cancer patients. These diets also reduced inflammatory levels.
Giordano et al., 2020 [14]	Evidence suggests that omega-3 PUFAs like DHA and EPA, found in unsaturated fats, may have anti-cancer properties and enhance treatment responses in breast cancer patients.
Heidari et al., 2020 [15]	Pre-diagnostic MD adherence was linked to lower mortality rates and improved prognosis in breast cancer patients, particularly in overweight or obese individuals. MD's effects on survival were more pronounced in certain age groups and aggressive cancer subtypes.
Long Parma et al., 2022 [16]	RCT findings indicated that the Mediterranean diet reduced co-morbidities, recurrence rates, and mortality among breast cancer patients, possibly due to its anti-inflammatory and antioxidant properties.
Montagnese et al., 2021 [17]	RCT results showed that omega-3 PUFA supplementation, a type of unsaturated fat, had positive effects on mitosis inhibition, inflammation reduction, and overall survival in breast cancer patients.
Negrati et al., 2021 [18]	Higher levels of β-carotene and lycopene, markers of adherence to the MD, are linked to a reduced risk of cancer, especially in breast cancer survivors. These biomarkers' antioxidant and anti-inflammatory properties suggest potential benefits for personalized nutrition in cancer prevention and management.
Theinel et al., 2023 [19]	Biomarkers like β-carotene and lycopene, associated with MD compliance, were linked to reduced cancer risk and served as indicators of dietary adherence. MD's antioxidant and anti-inflammatory properties play a role in preventing breast cancer.
Rein et al., 2022 [20]	RCT findings suggested that a diet rich in olive oil, a component of the MD, exhibited anti-cancer properties due to compounds like squalene and monounsaturated fatty acids present in olive oil.
Tawfik et al., 2023 [21]	RCT results demonstrated that dietary interventions combining unsaturated fats or MD components with conventional anti-tumor therapies showed promise in improving overall survival and reducing tumor growth in breast cancer patients.

Future research recommendations

In order to determine causal links between the Mediterranean diet or unsaturated fats and breast cancer outcomes, future research should concentrate on carrying out extensive, long-term, randomized controlled trials. To further our understanding, we should look into the molecular mechanisms and find biomarkers that predict responses to dietary interventions. To support personalized nutrition plans, research on the effects of diet, including genetic, epigenetic, and microbiome influences, is needed. Evaluation of effectiveness requires comparison studies with alternative dietary patterns and combination therapies. The cost-effectiveness of dietary interventions should also be evaluated, as well as behavioral factors influencing diet adherence in research. Nutritional advice for breast cancer patients can be more easily incorporated into treatment by creating clinical guidelines and providing training for medical professionals. 

## Conclusions

In conclusion, eating choices have a major impact on both managing and preventing breast cancer. A Mediterranean diet rich in unsaturated fats, particularly omega-3 polyunsaturated fatty acids, has been shown to improve quality of life, reduce death rates, and possibly even reduce the risk of recurrence in patients with breast cancer. Supplementing the diet with soy, legumes, fruits, vegetables, and foods rich in antioxidants, like olive oil, may also help to enhance the outcomes. Further research is required to fully understand the mechanisms underlying these benefits and to provide dietary recommendations tailored to individuals with breast cancer. Nevertheless, these findings highlight the necessity of an all-encompassing approach that includes dietary interventions to improve the prognosis and state of health of individuals with breast cancer. 
